# The downregulation of putative anticancer target BORIS/CTCFL in an addicted myeloid cancer cell line modulates the expression of multiple protein coding and ncRNA genes

**DOI:** 10.18632/oncotarget.20627

**Published:** 2017-09-02

**Authors:** Evgeny Teplyakov, Qiongfang Wu, Jian Liu, Elena M. Pugacheva, Dmitry Loukinov, Abdelhalim Boukaba, Victor Lobanenkov, Alexander Strunnikov

**Affiliations:** ^1^ Molecular Epigenetics Laboratory, Guangzhou Institutes of Biomedicine and Health, Guangzhou, China; ^2^ NIH, NIAID, Laboratory of Immunogenetics, Rockville, MD, USA; ^3^ The University of the Chinese Academy of Sciences, Beijing, China

**Keywords:** transcriptome, chromatin, CML, CTA, sncRNA

## Abstract

The *BORIS/CTCFL* gene, is a testis-specific *CTCF* paralog frequently erroneously activated in cancer, although its exact role in cancer remains unclear. BORIS is both a transcription factor and an architectural chromatin protein. BORIS’ normal role is to establish a germline-like gene expression and remodel the epigenetic landscape in testis; it similarly remodels chromatin when activated in human cancer. Critically, at least one cancer cell line, K562, is dependent on BORIS for its self-renewal and survival.

Here, we downregulate BORIS expression in the K562 cancer cell line to investigate downstream pathways regulated by BORIS. RNA-seq analyses of both mRNA and small ncRNAs, including miRNA and piRNA, in the knock-down cells revealed a set of differentially expressed genes and pathways, including both testis-specific and general proliferation factors, as well as proteins involved in transcription regulation and cell physiology. The differentially expressed genes included important transcriptional regulators such as *SOX6* and *LIN28A*. Data indicate that both direct binding of BORIS to promoter regions and locus-control activity via long-distance chromatin domain regulation are involved. The sum of findings suggests that *BORIS* activation in leukemia does not just recapitulate the germline, but creates a unique regulatory network.

## INTRODUCTION

Cancer-testis antigens are immunogenic proteins normally restricted to expression in the male germline but are frequently activated in various types of cancer [1-4]. Their immunogenicity, however, has become a less defining trait of members of this group, compared to the expression pattern, so the corresponding genes are also referred to as cancer-testis (CT) genes [5]. Despite a wealth of knowledge accumulated about CT genes, their biology in both cancer and in the normal germline is far from being understood. Particularly, areas such as their role as cancer drivers rather than biomarkers, their contribution to the stemness of tumor cells, and their molecular functions have yet to be fully addressed.

*BORIS/CTCFL* (Brother Of the Regulator of Imprinted Sites / CCCTC-binding Factor Like) [6] is a CT gene that is commonly activated in cancers [7]. BORIS is a paralog of *CTCF* (CCCTC-Binding Factor) [8], which has been branded a “master weaver of the epigenome” in multicellular organisms [9]. Indeed, CTCF is an inherently versatile and multifunctional DNA-binding protein, using 11 zinc fingers (ZF) to associate with a wide spectrum of target sequences [10]. CTCF has both a *cis* and *trans* modes of action: either directly controlling gene expression *via* enhancer or promoter binding in the first case [8, 11-20] or organizing chromatin domain compartmentalization and interstrand interaction in the second [18, 20-27]. Whilst the DNA binding domains of CTCF and BORIS are nearly identical and show indistinguishable DNA binding specificity *in vitro* [28-31] there is practically no similarity between the two proteins outside of the DNA binding domain. Thus, BORIS for quite some time was thought to be an alternative form of CTCF, functional only in the germline and in some cancers. A homozygous deletion of CTCF in mice resulted in early embryonic lethality [32], while BORIS knockout mice showed subfertility phenotype with an array of spermatogenic defects including a delay in gametogenesis and a reduction in testis size [28, 31]. Nevertheless, a recent study has revealed that BORIS emergence has likely resulted in the evolution of CTCF target sites CTSes themselves, so that BORIS binding preferentially occurs at clustered CTS, i.e. including two or more consensus DNA motifs in tandem [33]. Furthermore, clustered CTSes are able to bind both BORIS homodimers as well as heterodimers between CTCF and BORIS [33]. That breakthrough has resolved a nagging question on the mode of BORIS interaction with CTCF, firmly indicating that their relationship is mostly cooperative [33] rather than competitive [6, 31, 34] in cell types where both proteins are expressed. First of all, it was shown that BORIS binding is overwhelmingly associated with promoters and enhancers, i.e. substantially more specialized for direct transcriptional regulation compared to CTCF. Second, it was documented that cancer cells expressing high level of BORIS tend to display a characteristic pattern of BORIS binding to chromosomal locations, which is largely independent of the cancer cell origin, but instead recapitulates the binding landscape in male germ cells [33, 35]. Consistent with this interpretation, the ectopic expression of BORIS in MCF7 cells induced notable changes in the expression level of corresponding genes and recapitulated the germ cell pattern of BORIS binding.

K562 cells are chronic myeloid leukemia (CML) cells are dependent on BORIS, which is normally tightly repressed in soma [33], for the proliferation and self-renewal of stemness. Despite the progress in our understanding of BORIS as a broad spectrum transcriptional regulator, it still remains unknown what pathways result in the differentiation of these CML cells upon KO of the *BORIS* gene. Such knowledge is relevant in the context of anti-cancer differentiation therapy in particular, and for our general understanding of BORIS activation and its contribution to the self-renewal of cancer cells. The analysis of the gene expression in already-differentiated cells is not informative with respect to the identity of the events, downstream of BORIS suppression that triggered the differentiation. Thus, analysis of gene expression should be undertaken shortly after BORIS downregulation. We undertook a gene expression study using RNA-seq in order to identify potential pathways of immediate response to the downregulation of BORIS in K562 cells, which are dependent on BORIS for their proliferation.

## RESULTS

### BORIS downregulation results in a differential expression of genes in K562 cells

**Figure 1:**
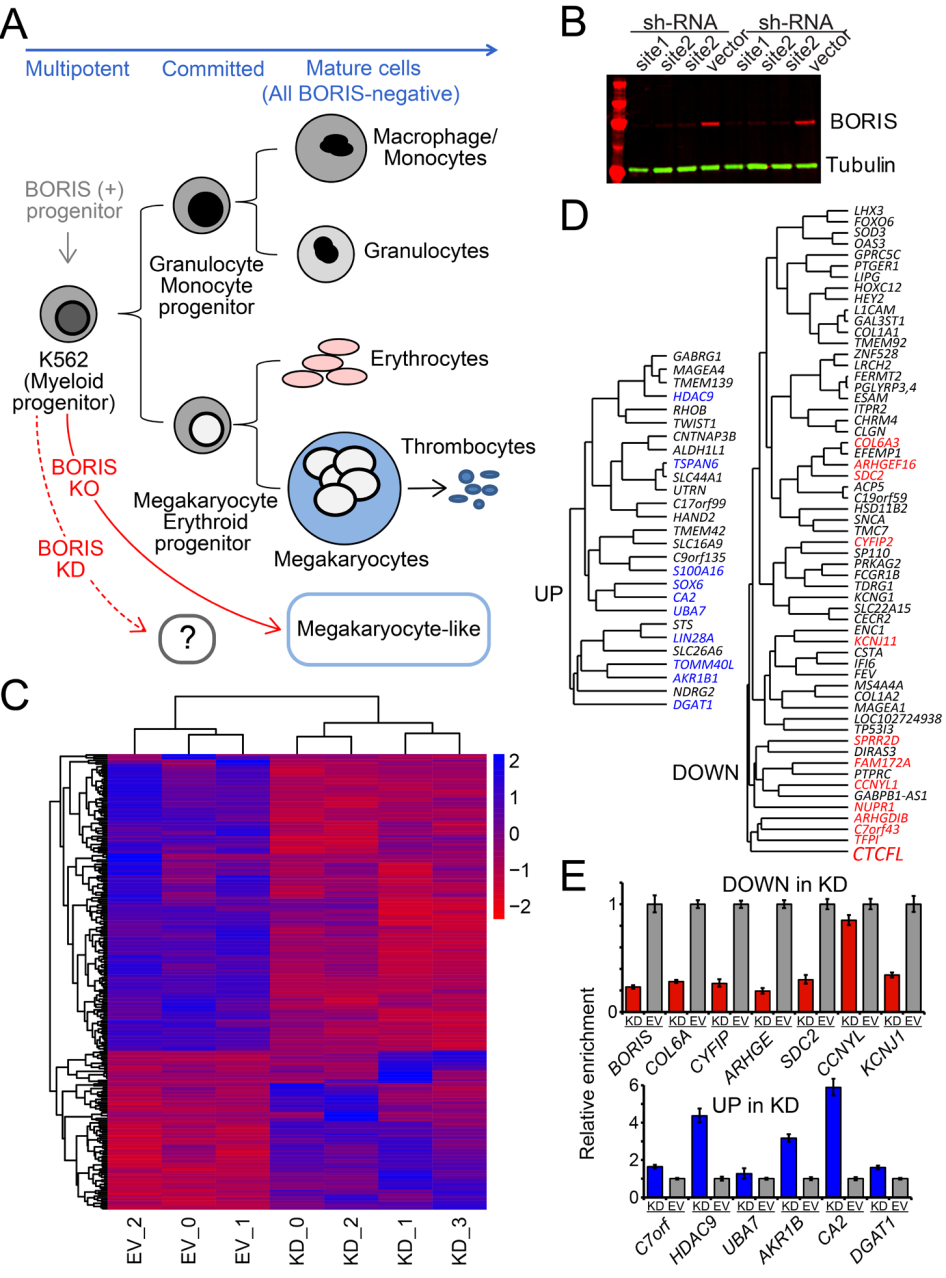
KD of *BORIS* leads to a limited but robust response in K562 transcriptome **A.** The differentiation pathways for K562 cells. K562 cells have properties of pluripotent precursors of normal hematopoiesis, and express both erythroid and megakaryocytic specific markers. The further differentiation of K562 along hematopoietic lineages could be triggered by specific treatments. This differentiation results in proliferation arrest, changed morphology and adhesion properties, as well as gain of protein markers specific for the terminal differentiation [146]. The KO of all transcribed copies of BORIS in K562 results in cells possessing a megakaryocyte-like morphology with a substantial number of corresponding markers activated [33]. As no intermediate steps were available for transcriptome analysis upon KO, the rapid downregulation of BORIS (KD) was analyzed in this work. **B.** Downregulation of BORIS protein upon knockdown. Immunoblotting shows reproducible depletion of BORIS protein over 10-fold (quantified by LI-COR) within 96 hours after shRNA induction, in two independent experiments with site 1 and site 2 shRNAs (see Methods). Anti-BORIS mAbs were as previously described [33], anti alpha-tubulin antibodies were used as loading control for quantification. **C.** A heatmap of the hierarchical clustering of differentially expressed genes based on mRNA-seq analysis. Only significant DE genes are shown. Blue - upregulated genes, red - downregulated genes. Both rows and columns are clustered based on their distance similarity. The color gradient corresponds to log2 of fold expression difference for each gene. EV- are control samples, KD_0 and KD_2 are shRNA-1 KD, KD_1 and KD_3 are shRNA-2 KD. **D.** The subset of hierarchical clustering of differentially expressed genes based on mRNA-seq analysis filtered for expression levels and fold change in expression. Shown in color are the robust DE genes, i.e. ones that displayed significant change even when datasets with lower than 4-fold downregulation of BORIS mRNA were included (only if the downregulation of *GAL3ST1*, a known direct downstream target of BORIS [28], was at least 1.5 fold). **E.** RT-qPCR validation of differentially expressed genes upon BORIS KD. Both downregulated and upregulated DE genes were normalized to the K562 cell line stably infected with the empty vector (EV) lentivirus.

Cell types possessing stemness, such as K562, can enter a differentiated state as a result of two concurrent processes: genes controlling the self-renewal become repressed, and the triggers of specific differentiation pathways are turned on. In case of K562 the two major differentiation pathways are into erythroid and megakaryocytic lineages (Figure 1A). With respect to gene regulation, both repression and activation could be potentially handled by BORIS, as we previously showed that it can act as either an activator or repressor of transcription, dependent on the particular gene [33, 36].

We knocked down BORIS in K562 cells to evaluate the immediate/early response, i.e. genes that change expression within 96 hours after KD induction. Typical downregulation of BORIS mRNA ranged from 2.5 to 4.5 fold. 12 knockdowns (KDs) were subjected to RNA-seq. The best four data sets with a > 4-fold downregulation of BORIS mRNA were taken into the downstream analysis. This corresponded to over 10-fold reduction of BORIS protein level (Figure 1B). As a control, three independent experiments were used, with K562 cells stably infected by the corresponding empty lentivirus vector (EV) and induced by doxycycline. RNA-seq samples showed good correlation between the replicates (based on Pearson correlation) and robust changes in gene expression in response to BORIS KD (Figure 1C). The datasets with lower degrees of BORIS mRNA depletion, while excluded from subsequent analyses, were nevertheless utilized to pinpoint the most robust DE genes (shown in color in Figure 1D).

Chronic myeloid leukemia is commonly triggered by a chromosome 9 to 22 translocation that generates a fusion of the *BCR* (Breakpoint Cluster Region) and *ABL* encoding a tyrosine kinase. Incidentally, BORIS does have a strong binding at the *BCR* promoter region in K562, yet the *BCR* locus is not differentially expressed in KD. This indicates that the regulation of *BCR-ABL1* transcript is unlikely to be responsible for K562 proliferation inhibition upon BORIS KO [33]. Therefore, some alternative and/or downstream pathways must be responsible. In the RNA-seq data, 149 genes were significantly downregulated upon BORIS KD (Cufflinks *q*-value < 0.05, log2(fold) > 0.4), and 85 were significantly upregulated (Cufflinks *q*-value < 0.05, log2(fold) < -0.4) (Supplementary Table 1). A more stringent cutoff, i.e. the expression level of higher than 0.5 FPKM and the minimal DE ratio of two-fold resulted in 20 up and 54 down genes, which are discussed below. A subset of these genes (10 upregulated, and 12 downregulated excluding *BORIS*, in color in Figure 1E) had significant DE even when datasets with lower *BORIS* downregulation were included in the analysis (*BORIS* mRNA between 2 and 4 fold, and *GAL3ST1* over 1.5-fold downregulated). These genes may be prime candidates for the most responsive genes upon BORIS downregulation.

Upon the comparison of BORIS KD DE genes to the RNA-seq results of KO in [33] it was apparent that the datasets are overlapping only slightly, and the similarly affected genes, were not particularly revealing with respect to the trigger of the differentiation in KO (Figure 1A). Out of three upregulated genes in common, i.e. *IL10RA, PASD1,* and *SLC44A1,* only *PASD1* may be relevant, as it is a potent regulator of transcription and a CT gene itself [37, 38]. The commonly significantly downregulated genes, i.e. *COBL, COL1A2, EFEMP1, LRCH2, MS4A3, MS4A4A, OAF*, and *SDC2*, do not contain regulatory proteins, apart from BORIS itself. This outcome, however, could have been expected, as the majority of gene expression in cells that spontaneously differentiated into megakaryocytic lineage upon stable BORIS downregulation in KO would have been too distant/downstream from the initial phase of the BORIS downregulation, and thus unlikely to correspond to the first-response genes.

One could assume that the genes with BORIS binding in *cis*, e.g. at the promoters, are most likely to be the direct targets of BORIS regulation. However, it was evident that among DE loci, the genes with BORIS binding in the immediate promoter’s vicinity were approximately evenly represented with ones without any BORIS peaks there. The latter class likely reflects the control of gene expression by BORIS *via* enhancer activity and/or in general folding of chromatin domains in K562 [33].

### Genes upregulated upon BORIS KD

**Figure 2:**
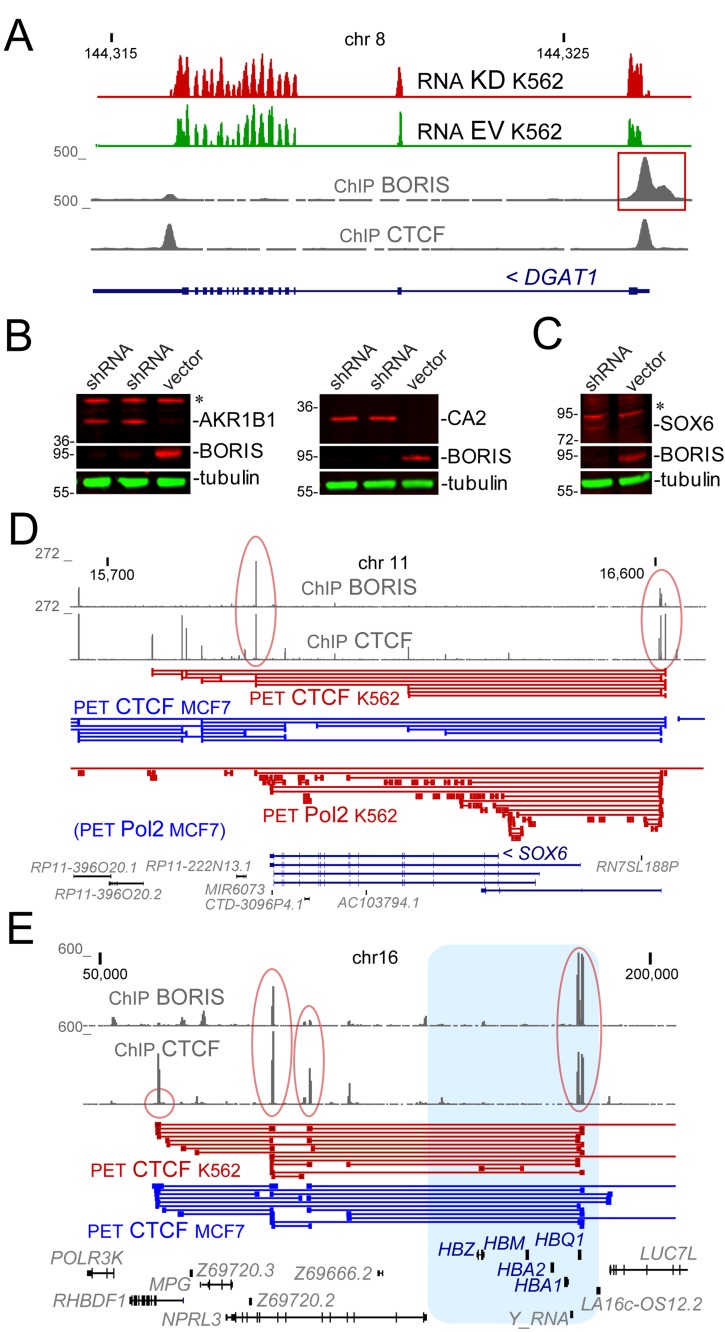
The examples of genomic loci and proteins that were upregulated upon BORIS KD **A.** The genomic structure of *DGAT1* locus. The RNA signal (TopHat alignment) shows the comparison of KD and EV control. The ChIP signal [33] shows the sites for CTCF and BORIS binding. The BORIS peak at the transcription start site is boxed in red. Numbers on the top show chromosomal coordinates (in kb), and numbers on the left indicate the range of data-view signal scaling (in Genome Browser arbitrary units). **B.** The upregulation of AKR1B1 and CA2 proteins upon BORIS knockdown. Immunoblotting shows the depletion of BORIS in two independent experiments and the appearance of the corresponding upregulation of AKR1B1 and CA2 protein in the whole cell extracts 96 hours after shRNA induction. Alpha-tubulin is a loading control. Molecular mass markers are shown on the left (x1000). **C.** The upregulation of SOX6 protein in chromatin fraction upon BORIS knockdown. Immunoblotting shows the depletion of BORIS in the whole cell extracts and the appearance of the corresponding SOX6 protein 96 hours after shRNA induction. Molecular mass markers are indicated on the left (x1000). **D.** The *SOX6* spanning region in human genome with the corresponding CTCF and RNAP II (Pol2) ChIA-PET data [46]. The BORIS and CTCF peaks corresponding to two major anchor sites for the locus are circled in red. Numbers on the top and on the left are as in **A.**. **E.** The globin locus. The hemoglobin genes are shown in blue. The ChIP signal pileup shows the two anchor sites for the globin locus (circled in red), with one on the right including a BORIS and CTCF cluster site.

It seemed likely that the DE genes involved in the cell cycle would have been the primary candidates for how BORIS controls the cascade that eventually results in cell differentiation. Nevertheless, the *STS* gene, with the strongest relative upregulation , encodes a steroid sulfatase, which is involved in hormone metabolism. However, the upregulated transcription was limited to a longer transcript or a minor isoform originating from an alternative promoter, which might indicate that it represents a regulatory transcription, possibly germline-related. Indeed, the corresponding RNAP II binding in K562 was limited to the immediate vicinity of BORIS peak at this site (not shown). This transcription could also be related to the fact that *STS* is a gene that escapes X-chromosome inactivation [39]. Among other genes that were clustered with *STS* (Figure 1D) were several genes with ubiquitous expression in the normal tissues and documented overexpression in cancers: *SLC26A6*, *TOMM40L*, *AKR1B1*, *NDRG2* and *DGAT1* (based on TCGA data). All of them have non-zero expression in K562 and have BORIS binding in their promoters, which likely results in their upregulation upon BORIS KD, as exemplified in Figure 2A, with *DGAT1*. However, these genes do not seem to be obviously linked to K562 differentiation. Nevertheless, sampling of protein expression, e.g. *AKR1B1* (Figure 2B)*,* by immunoblotting showed that protein upregulation could be readily detected.

Functionally, one gene did catch our attention: *LIN28A* [40] is a potent pluripotency factor [41, 42] and a marker of cancer stem cells [43-45]. Thus, it could link BORIS to powerful pathways of mRNA and miRNA regulation, as LIN28A is an RNA-binding protein [40]. Nevertheless, *LIN28A* does not appear to be regulated by BORIS in *cis*, as the closest strong BORIS binding peak [33] is 80 kb downstream, and there is no BORIS/CTCF-anchored chromatin domains enclosing it [46].

Another set of genes clustered together (Figure 1D) included four genes, *UBA7, CA2, SOX6* and a rather uninformative *S100A16*. CA2 has a strong BORIS&CTCF binding site in the promoter and encodes a carbonic anhydrase. It is widely expressed in many tissues, including erythroid lineage [47, 48]. Thus, its activation in BORIS KD K562 (Figure 2B) could be a sign of commitment to the differentiation. The two remaining genes are more insightful. In the corresponding KO mice, the *UBA7* homolog, *Ube1L,* was shown to be directly involved in the regulation of the transplantation proficiency, i.e. proliferation, of hematopoietic progenitors [49]. Incidentally, the *UBA7* gene does not have BORIS bound at the promoter in K562 cells, indicating that the BORIS-mediated regulation is exerted indirectly, possibly by rearranging 3D chromosomal structure like CTCF [33]. Indeed, while the *UBA7*-containing region is involved in multiple interactions as shown in ChIA-PET (Chromatin Interaction Analysis by Paired-End Tag/Sequencing) data [46], there is a *UBA7* locus-specific CTCF loop, with one anchor enriched by BORIS, while the other is occupied by CTCF only, with corresponding strong RNAP II-specific interactions present only in BORIS-positive K562, but not in MCF7 (Supplementary Figure S1).

Another gene clustering with *UBA7* is the transcription factor *SOX6* (Figure 1D). With concentrated extracts, the expression of protein itself is detectable in BORIS KD (Figure 2C). The *SOX6* gene is large, spanning almost 500 kb, with strong BORIS binding sites both upstream and downstream of the locus. The main promoter region of *SOX6* is not occupied by either BORIS or CTCF in K562 (Figure 2D). This could reflect the fact that the transcriptional control by BORIS and CTCF at the *SOX6* locus is mediated by these proteins *via* a larger chromatin domain. The CTCF ChIA-PET data [46] indicates that the *SOX6* locus loop is fully enclosed between CTCF sites regardless of BORIS presence (Figure 2D) . Nevertheless, the regulatory potential of BORIS might be exerted through anchor sites in cooperation with CTCF (Figure 2D). Indeed, the RNAP II ChIA-PET data [46] indicate that there is a lot of RNA polymerase-dependent interactions within *SOX6*, many of them anchored at BORIS bound sites, in BORIS-positive K562, but not in MCF7 (Figure 2D). *SOX6* overexpression is known to induce differentiation in several cell types [50-54].

Among other genes in the DE upregulated group, which could be considered informative, is *TWIST1*. Its overexpression has been directly implicated in both CML [55] and AML [56] biology and in epithelial-mesenchymal transition [57]. It provides an additional argument that the highest amplitude effect upon BORIS KD could involve a larger chromatin domain control, rather than a direct promoter regulation. Indeed, the locus identified in RNA-seq as corresponding to *TWIST1* (chr7.14) in fact overlapped with another upregulated gene *HDAC9* [58]. Both genes are virtually silent in K562 in the presence of BORIS. Incidentally, co-regulation of *Hdac9* and *Twist1* genes has been reported in mice and was attributed to having a common enhancer within the *Hdac9* intron [59].

### Pathways upregulated upon BORIS KD

When assessing the group of differentially upregulated genes as a whole, without focusing on the individual top scoring genes, one can pinpoint some indicative pathways upregulated by BORIS KD (Supplementary Table S2), despite the relatively small number of DE genes in our case. The most telling, notwithstanding the low expression, is a set of hemoglobin genes, *HBA1, HBG1, HBG2, HBA2, HBZ, and HBE1*. It is an additional indication of some BORIS involvement in blocking of a differentiation pathway, erythroid in this case, in K562. It also appears likely that BORIS is involved in the control of the whole hemoglobin locus (Figure 2E), as it was established for CTCF [60-62]. Incidentally, both *ZBTB7A/POK*, which has a defining role during erythroid differentiation and in cancer [63, 64], and *BCL11A* involved in hemoglobin switching [65, 66] both have strong BORIS binding upstream of their TSS [33]and were also moderately upregulated in the present study. At the same time, genes that are required for hemoglobin assembly, such as *AHSP*, were unaffected by BORIS KD.

Among the upregulated pathways, there were also several prominent transcription factors and cofactors. These factors are controlling substantial networks of downstream genes and involved into morphogenetic and/or differentiation processes: *GDF,* as well as *HAND2* and *FOXA2*, both prominent early mesoderm genes. *HAND2* has a BORIS site at its promoter and is known to be aberrantly methylated in CLL [67], which may indicate an activity of BORIS counteracting aberrant hyper methylation, similar to *TP53TG1*. While HAND2 was shown to be a player in differentiation and trans-differentiation [68], it shows low expression in K562 and a relatively minor increase. *GDF2/BMP9*, encoding a TGF-beta superfamily member [69], and *FOX2A*, on the other hand, do not have BORIS binding at its promoter, and are upregulated less than twofold.

The group of signaling-related and cytokine-related genes also included genes that either did not (*OSGIN1* and *HYAL1*) or did (*CA2, SLC26A6, CHRD, RHOB* and *APBB2*) have BORIS binding in the immediate promoter vicinity. Some of these genes could negatively regulate cell growth, such as *OSGIN1* [70]. Published data on *HYAL1* are somewhat contradictory with respect to its effect on the proliferation of cancer cells [71, 72]. *RHOB* is probably most interesting in this group. It encodes a small GTPase with a direct link to apoptosis regulation [73]. Furthermore, the mechanism of its involvement is likely mediated by the binding to TP53 isoforms and thus connects to the very core of the ability of cancer cells to proliferate [74].

### Genes downregulated upon BORIS KD

As was mentioned, one could expect to find potential genes involved in the maintenance of self-renewal of the K562 cells among the genes that are downregulated upon BORIS KD. Indeed, among genes with highest DE (Figure 1D) several genes did stand out. Among the genes that tightly clustered with *BORIS* or formed the next cluster (Figure 1D), only *TFPI* and *PTPRC* were assigned to a biological process pathway (Supplementary Table S3). The rest, i.e. *C7orf43*, *ARHGDIB*, *NUPR1*, *GABPB1*-*AS1*, *CCNYL1*, *FAM172A*, *DIRAS3*, and *SPRR2D* provide only indirect hints on the potential BORIS facilitation of K562 proliferation and stemness self-renewal. The highest individual fold change in this group was observed for *FAM172A*. which remains a poorly characterized gene [75-77] The *CCNYL1* gene is more informative, with the second highest fold downregulation in BORIS KD and BORIS binding in its promoter. It is a CDK6 cyclin, which is normally specific for germ cells [78].

In general, at present we could only infer some relevance of most genes in the set to BORIS biology in K562. The KD of *NUPR1* was shown to inhibit both cell proliferation and migration and resulted in a cell cycle arrest in more than one cancer types [79, 80]. *NUPR1* is a chromatin-bound regulatory protein [81] that interfaces with both DNA methylation and histone acetylation genome-wide [82, 83]. The *DIRAS3* gene encoding a member of the *ras* superfamily presents a special case with respect to BORIS-mediated regulation, as the expression of this gene is tightly controlled epigenetically; it is expressed monoallelically due to maternal imprinting [84]. *SPRR2D* is a part of a cluster of several small proline-reach proteins encoding genes on chr1(q21.3), which was identified as epidermal differentiation complex [85]. The cluster is enclosed into a chromatin loop with one of the CTCF anchors occupied by BORIS, according to ChIA-PET data [46]. *CYFIP2*, which has a very strong BORIS binding around the TSS, not just in K562 but as well in all other BORIS-positive cells analyzed by ChIP in [33], is directly linked to the actin metabolism and the fragile X syndrome *via* interaction with FMR1 protein [86]. There are also genes that are known to directly control transcription and chromatin structure: *FEV, CECR2, ZNF528, HEY2, FOXO6* and *CECR2.*

### Pathways downregulated upon BORIS KD

GO analysis showed that 42 of downregulated genes fell into clusters corresponding to defined biological pathways. Due to the small number of downregulated genes, pathway discovery was not particularly informative with respect to the role of BORIS in K562 self-renewal or regarding triggering the differentiation upon its downregulation. Nevertheless, it was noteworthy that the largest group of downregulated genes was the group (12 genes in total) related to blood coagulation (*L1CAM, ESAM, ITPR2, KIF5A, FN1, SERPINC1, SYK, TFPI, CD9, COL1A1, COL1A2, DOCK11*) (Supplementary Table S3). The coordinated downregulation of this group by BORIS could indicate that one way BORIS interjects itself in the control of myeloid cancer cells expression program, is by controlling the expression of megakaryocytic pathway genes. Incidentally, some other hematopoiesis-related genes and pathways were also evidently regulated by BORIS, which could reflect a broad spectrum of K562 cells’ dependency on BORIS [33]: *L1CAM*, *PTPRC*, *SYK, PGLYRP4, PGLYRP3, PTPRC,* and *SYK* (Supplementary Table S3).

### Multiple miRNAs change their expression upon BORIS KD

**Figure 3:**
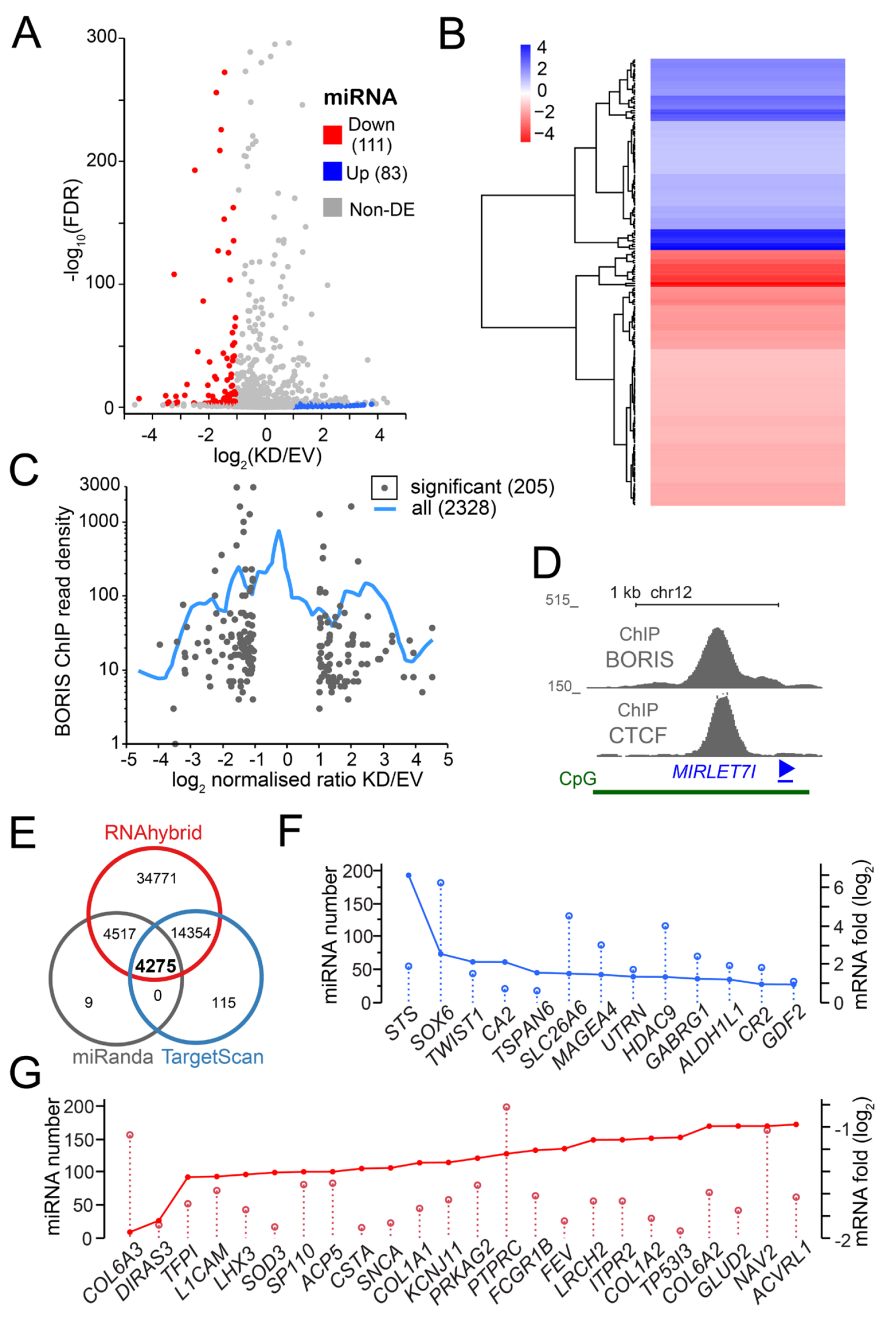
The RNA-seq analysis of miRNA upon BORIS depletion **A.** A volcano plot of all differentially expressed miRNA. Each dot corresponds to a potentially differentially expressed miRNA, with colored corresponding to significant DE MiRs and grey to ones under significance threshold, as described in Methods. **B.** A heatmap of the hierarchical clustering of differentially expressed miRNA based on RNA-seq analysis. Blue - upregulated genes, red - downregulated genes. **C.** A plot representing the relationship between the differential expression of miRNA and the read density from BORIS ChIP-seq [33], i.e. number of sequencing tags per 2 kb window centered at a given MiR-encoding genomic sequence. The blue smoothing line represents the whole dataset of expressed MiRs converted into genomic coordinates, the dots correspond only to significant DE MiRs. **D.** *MIRLET7I* locus structure, with ChIP [33] and ChIA-PET [46] data combined. **E.** Target analysis of DE miRNA. Vent diagram of potential miRNA regulated targets shows the overlap of all three methods used, with corresponding numbers of target sequences. **E.** The overlap plot of a sample of upregulated mRNA with the corresponding miRNAs expressed in K562. **F.** The overlap plot of some significantly downregulated mRNA with the corresponding K562-expressed miRNAs. **G.** The overlap plot of significantly upregulated mRNA with the corresponding K562-expressed miRNAs.

As was shown above, BORIS displayed a multifaceted involvement in the regulation of downstream genes, including long- non-coding RNAs: *TP53TG1* [87] and *LOC102724938*. The role of BORIS in the expression of small noncoding RNAs has never been studied. Therefore, we conducted a series of four additional RNA-seq experiments aimed at the identification of short ncRNA that would be differentially expressed upon BORIS KD, and focused on the ones that are relevant to gene expression regulation, namely miRNA and piRNA, for downstream analyses. While several surveys were made of the miRNA repertoire in CML [88-90], the miRNA molecular makeup of these cancers is far from completely understood. Thus, the goal of this analysis was to identify miRNAs, which change their expression and are known to be associated with the inhibition of the proliferation of cancer cells, and CML in particular. In our miRNA-seq data, 83 were up-regulated *versus* 111 down-regulated (Figure 3A, 3B), which included a number of uncharacterized/novel miRNA (Supplementary Table S4).

In order to get insight into the putative mechanism of BORIS control of miRNA expression we assessed the relative enrichment for BORIS binding in the vicinity of miRNA genes. The Figure 3C shows that the highest density of BORIS biding (ChIP) is not associated with the genes displaying the most extreme DE. This indicates that in K562 cells, similarly to protein-coding genes, BORIS is also acting in *trans*, *via* enhancers and/or 3D chromatin domain folding. For example, let-7i-5p is a part of a CpG island encompassing the TSS of the *LINC01465* gene with a very strong BORIS and CTCF binding (Figure 3D). let-7a-3p, on the other hand, is expressed in an intergenic region harboring several ncRNAs and flanked by strong BORIS and CTCF binding sites, indicating a potential regional control of its expression by BORIS, while miR-142-5p is located in a 15-kb region expressing several noncoding RNA, without obvious binding of BORIS in the vicinity. Finally, the miR-1296-3p MiR is intragenic to the *JMJD1C* gene that has a strong BORIS binding site in its promoter, with a similar situation observed for miR-340-5p (the *RNF130* gene), and for miR-224-3p (*GABRE* gene). Mature sequence miR-7-5p is encoded at three different loci in human genome, none of which appear to be associated with BORIS binding in cis.

Among miRNA downregulated upon BORIS KD, miR-7-2-3p and let-7a-5p stand out with highest fold (Supplementary Table S4). miR-7-2-3p information is presently limited to biomarker surveys [91, 92]. The let-7a-5p expression is normally maintained at a relatively stable level [93], and the three loci that encode it have no direct association with BORIS binding in K562. Nevertheless, the *MIRLET7A2* locus is located within a nearly megabase region expressing several ncRNAs, which is flanked by strong BORIS and CTCF binding sites.

### Multiple downstream pathways are regulated by miRNA under BORIS control

**Figure 4:**
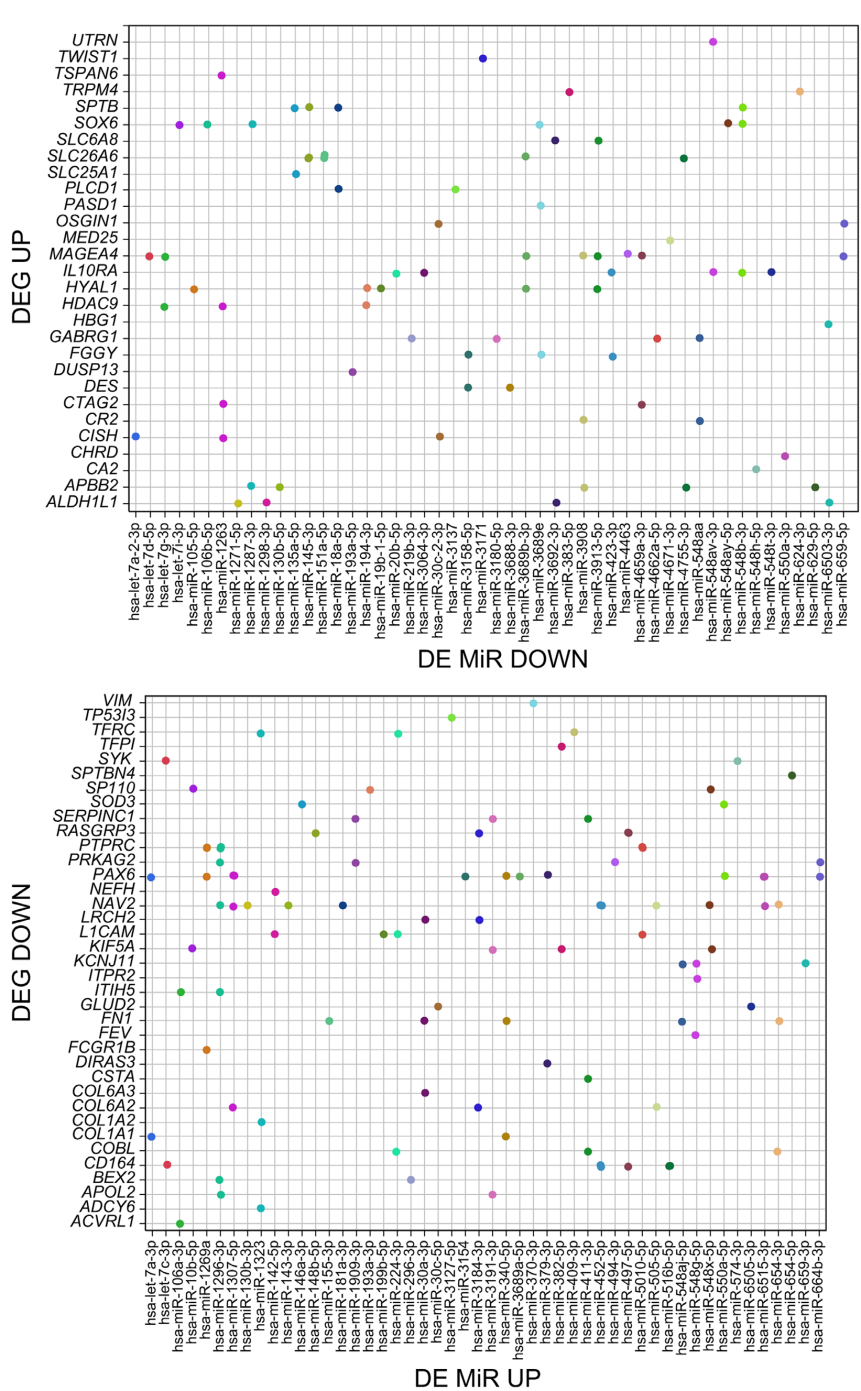
The correspondence between the significantly differentially expressed mRNAs and MiRs upon BORIS KD The dots represent the individual significant DE MiRs regulating a given gene, but expressed in a reciprocal fashion, as indicated in panel titles.

The chief value of miRNA analysis in our case was to have a measure of the regulatory reach by BORIS, mediated by miRNA expression. We used several tools (see Methods) to identify the targets of differentially expressed miRNAs after filtering for significance level. As all these methods are known to be prone for false-positive identification, we only focused on 4275 predicted by all three methods (Figure 3E). This still indicates that BORIS has a potential to control a sizeable genetic network in these BORIS-positive CML cells. As the network of DE miRNA resulting from BORIS KD is highly interconnected, with multiple pathways potentially affected (Supplementary Figure S2), it is a challenging task to extract the actionable components. One measure of the affected nodes of regulation that are relevant to the eventual K562 differentiation is the links between the DE mRNA and the DE ncRNAs, which are known/predicted to affect the corresponding mRNAs. Indeed, for most DE mRNAs there were from a dozen to a couple of hundred potential miRNA that were expressed in K562 (Figure 3F, 3G). While the compound effect of multiple miRNAs regulating the same gene but falling under a threshold of significance is not easy to evaluate, focusing on the pairs of significant DE MiRs and significant DE genes that were reciprocally changed (miRNA up with mRNA down, and vice versa) enables one to generate a snapshot of the putative regulatory network involved (Figure 4). This could also provide a plausible explanation for some genes with a changed expression, for which we don’t detect BORIS binding in the promoter vicinity or at the base of enclosing chromatin loop.

### A number of piRNAs are regulated by BORIS in K562

**Figure 5:**
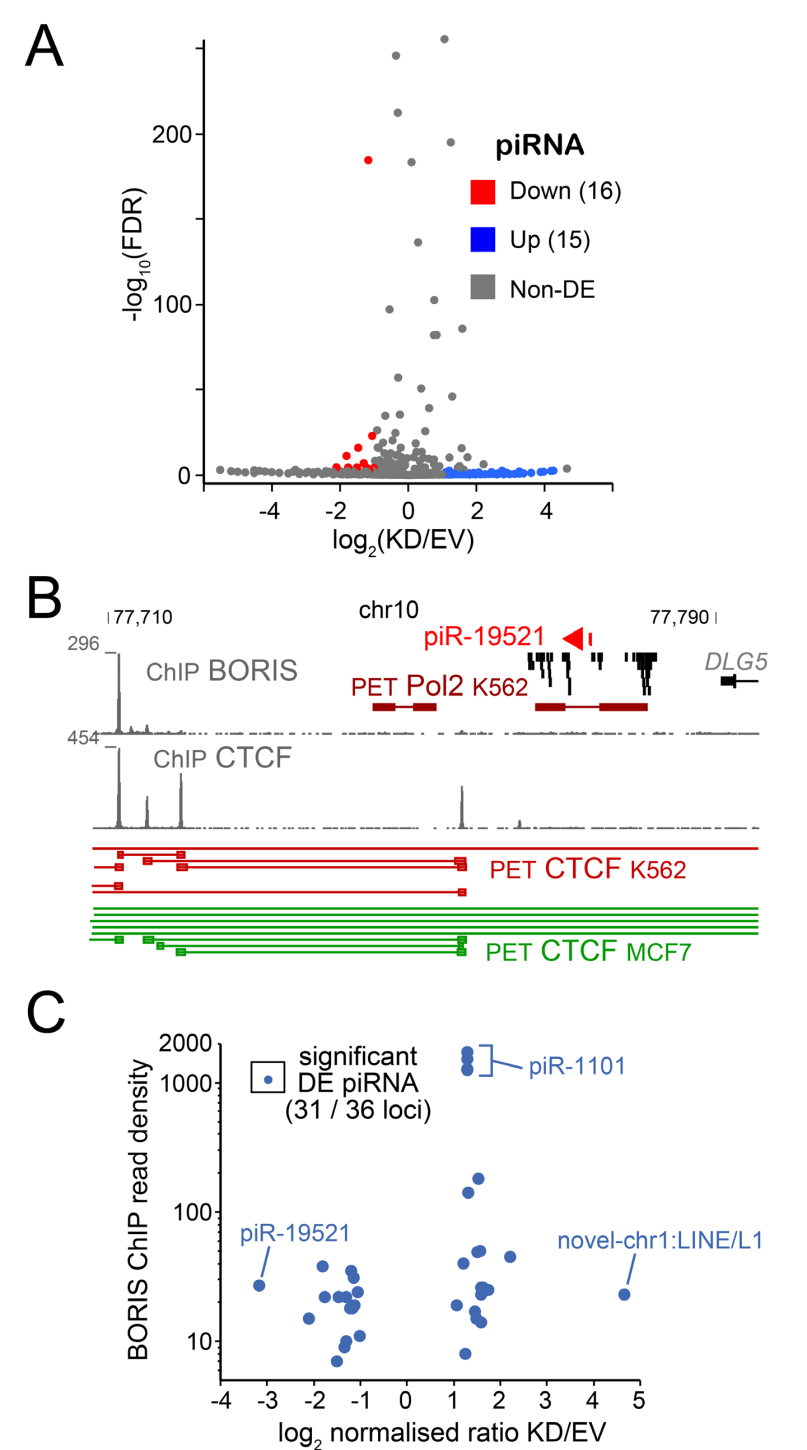
The RNA-seq analysis of piRNA **A.** Volcano plot of K562-expressed piRNA shows the relationship between fold change of expression and p-value. **B.** The structure of *pIR19821* locus, with BORIS and CTCF ChIP data in K562 combined with CTCF ChIA-PET data from K562 (BORIS-positive) and MCF7 (BORIS-negative) [46]. C. The differential expression of piRNAs in relation to BORIS ChIP (read density) from [33], with some piRNA examples indicated.

Piwi-interacting RNA (piRNAs) are small non-coding regulatory RNAs that are abundantly present in testis [94, 95]. piRNAs are also involved in the control of transposable element (TE) expression [96]. We have previously showed that BORIS acts as a co-repressor for at least one TE family, SVA [36], however BORIS has not been implicated in regulating piRNA expression. Therefore, it was intriguing to see whether BORIS positively regulates piRNA expression in cancer cells, presumably by analogy with the spermatogenic germline. Upon analysis of the small ncRNA-seq data we identified 16 piRNA that were upregulated and 15 - downregulated (Figure 5A), which corresponded to 36 genomic locations. Only one known piRNA that showed a robust downregulation upon BORIS KD, piR-19521, was expressed in a large cluster of ncRNAs with no obvious genomic landmarks potentially linking its expression to BORIS (Figure 5B). However, the 3D structure of the corresponding locus in K562 is different from the BORIS-negative MCF7 cells, both for CTCF and RNAP II - mediated organization (Figure 5B). This may be due to the presence of BORIS in the K562 cells, but additional dissection of the pathway could be required to establish a specific link. In general, for all the DE piRNAs, there was little coincidence with BORIS binding in the immediate 2-kb window (Figure 5C). Following are known piRNAs that stand out with respect to their higher expression and fold of upregulation: piR-586 expressed from the same strand as several long noncoding RNAs (*DGCR9* and *DGCR5*), which have strong BORIS binding peaks; piR-18780 overlapping with a *MIR3* repeat; piR-1101 with several genomic locations corresponding to *LINE1*; and the piR-018569, a part of the last exon of the *GLG1* gene, which has BORIS binding in the promoter. The information on novel piRNAs is included in Supplementary Table S4, with most of them originating from TE sequences.

Thus, it appears that piRNAs follow the same pattern of regulation by BORIS that was observed for mRNAs and miRNAs, i.e. while BORIS binding in cis is likely playing a role, the regulatory reach of BORIS *via* long-range interactions in the chromatin seems to be more dominant.

## DISCUSSION

As a result of this work, we demonstrated that BORIS/CTCFL is controlling a sizeable genetic network in cancer cells. The K562 is the first cancer cell line that was shown to be addicted to a CT gene. Namely, the KO of the, normally testis-specific, *BORIS* gene resulted in a terminal differentiation of K562 cells [33]. At the same time, the transcriptome of the K562 differentiated after *BORIS* KO [33] did not enable the elucidation of the actual role played by BORIS in the cancer cell line itself, because cells differentiated and hence lost their potential to proliferate and self-renew. While *BORIS* is the CT gene that is most commonly activated in cancers among other CT genes [7], most cancers, unlike K562 cells, have low level of BORIS expression. Therefore, it was especially important to get inroads into understanding of the mechanism of K562 cells addiction to BORIS. The present detailed analysis of fast BORIS downregulation is helpful in better understanding the scope of CT-mediated regulation in cancer cells, as well as it insightful with respect to the differentiation therapy of CML.

In the present work, a nearly 5-fold rapid downregulation of main *BORIS* transcript by shRNA, which resulted in over tenfold reduction in BORIS protein levels, was shown to affect the expression of numerous transcripts, including both mRNAs and ncRNAs. That enabled us to survey the early response to BORIS depletion, and to pinpoint prospective pathways that could become dependent on BORIS in cancer. The normal development, differentiation, and maturation of precursors for blood cells are, essentially, an interplay between two reciprocal processes: the eventual loss of their self-renewal potential, and a sequential gain of gene expression patterns characteristic for specific lineages. While K562 cells are tumor cells, they, in a typical fashion for CML, retain the capacity to differentiate and mature (Figure 1A). Thus, one is to expect a similar interplay of genetic networks upon commitment to differentiation. One surprising result of the significant downregulation of BORIS in this work is, undoubtedly, the fact that relatively few genes were affected. It is especially striking, if one considers that the number of BORIS binding peaks in K562 runs in tens of thousands [33]. This inevitably leads to a conclusion that, while BORIS activation establishes epigenome-wide pattern of binding in a germline-like fashion [33, 35], the actual addiction of K562 to BORIS is restricted to a very limited number of pathways.

Among the upregulated mRNAs, a clear connection was evident to the genes that could potentially inhibit proliferation. Namely, *SOX6, LIN28A, HDAC9* and *UBA7* all directly interface with the proliferation control. *SOX6* can suppress cell proliferation and induce differentiation upon overexpression in several model systems [50-53] including erythropoiesis [54]. The link of *SOX6* dosage to differentiation potential is also bidirectional, as insufficient *SOX6* can prevent differentiation [97]. Thus, it is not surprising that *SOX6* transcription is tightly regulated by a negative auto-regulation loop [98]. Furthermore, it was already directly shown that ectopic *SOX6* overexpression induced erythroid differentiation within 9 days in both K562 and primary erythroid cultures from human blood [99]. Thus, *SOX6* is a suitable candidate for a strong downstream effector of BORIS KD that might be a part of the response to BORIS downregulation leading to cell differentiation.

LIN28A blocks the maturation of let-7 miRNA [100, 101] and so regulates a number of downstream cell cycle proteins including cyclin D1 (CCND1) and CDC25A. Furthermore, LIN28A also directly regulates several mRNAs leading to reprogramming of cellular metabolism [102] and may also control gene expression directly *via* epigenetic remodeling of promoters [103]. Thus, LIN28A could be one of the key factors enabling reprogramming in K562 upon BORIS KD. The *UBA7/UBE1L* gene was shown to reduce cyclin D1 protein levels, and it possibly affects other pathways *via* increasing ISGylation, thus having a pronounced anti-proliferation effect in cancer cell lines [104, 105]. *HDAC9* is a histone deacetylase-like protein, which could be a significant factor in determining some of the downstream epigenetic effects of BORIS suppression in K562 acting through modulating transcription as other class IIa HDACs [106]. There is ample evidence directly linking *HDAC9* to oncogenesis [107, 108, 109], including lymphomas [110]. Most recent data also indicate that overexpression of *HDAC9* could directly interfere with cell cycle by inhibiting cyclin D1 expression *via* cooperation with PC3/Tis21 [111].

All four genes directly feed into the inhibition of cyclin D1 protein or the encoding gene, which is a common proto-oncogene. The corresponding *CCND1* gene is known to cause malignant cell transformation in both CML and AML upon IGH fusion [112] and upon overexpression/dysregulation in other cancers, even in the absence of other major oncogenic hallmarks, such as p53, MYC and RAS [113]. However, the *CCND1* mRNA level ends up not significantly changed upon BORIS KD, indicating the complexity of the regulation involved. One of the possible counterbalances could be a very high expression of *CDC25A*, regardless of the BORIS KD. Nevertheless, *SOX6* is one of the most suitable candidate downstream BORIS, for inhibiting K562 proliferation upon BORIS KD. This gene is known to trigger differentiation in a number of systems [52, 53, 98]. The fact that BORIS regulates this gene in K562 cells is likely attributed not to the direct control of its promoter, but rather *via* invasion of the chromatin loop enclosing only the *SOX6* locus and normally maintained by CTCF in somatic cells. This could be a reflection of the specific regulatory circuit potentially controlled by BORIS not just in myeloid progenitors, but also in other cancer cells that activate BORIS.

We also observed clear signs of changed regulation of a certain number of protein-encoding genes relevant to erythrocyte and megakaryocyte differentiation pathways, as well as a number of testis-specific genes, in response to BORIS KD. These finding shows that the involvement of BORIS in the maintenance of self-renewal and stemness of K562 is multifaceted, but still rather limited in scope, considering the abundance of BORIS binding to chromosomes.

Among the genes that were downregulated upon BORIS depletion we see a coordinated downregulation of some progenitor-specific genes as well as decreased expression of some proliferation factors, e.g. *NUPR1*, as well as genes that are normally germline specific (such as *CCNYL1*), but are activated in tumor cells owing, at least in part, to BORIS expression. *CCNYL1* was recently identified as a germline-specific cyclin for CDK16 [78], and thus appears to be a bona fide testis target activated by BORIS, possibly representing an additional link to cell cycle controls that are facilitated by BORIS in K562 cells.

The other genes involved in the control of transcription or chromatin remodeling are *FEV*, *CECR2, ZNF528, HEY2, and FOXO6*. Of those, *HEY2* acts upstream of Notch in blood lineages differentiation [114] and its KO in mice results in severe and early development defects [115]. Another interesting gene here is *CECR2* encoding a bromodomain protein, which was implicated in chromatin remodeling and has a strong morphogenetic function in spermatogenesis [116].

In conclusion, as a result of surveying the mRNAs differentially regulated upon BORIS KD, it is evident that there are signs of both inhibition of proliferation and the commitment to differentiation. At present, it is unclear, what is the threshold of BORIS expression that becomes physiologically meaningful in cancer cells. As was demonstrated here, the nearly complete depletion of BORIS protein, still did not trigger K562 differentiation, despite showing the signs of cell commitment. This agrees well with low, even if pervasive, BORIS expression in tumors [117, 118], and indicates that the dependence of K562 on BORIS is not embodied by numerous pathways.

One possible mechanism that prevents BORIS from changing expression of thousands genes in K562 cells instead of hundreds observed, is some additional level of regulation that might work as feedback regulation. Non-coding RNAs in general, and micro-RNAs in particular, could provide such a mechanism. In this work, for the first time, we evaluated an involvement of BORIS in the control of the expression of small noncoding RNA. While it was hinted by the DE of some long noncoding RNAs (lncRNAs) upon mRNA-seq analysis, the revealed scope of BORIS involvement in short RNA expression is rather far-reaching, with thousands of downstream genes potentially affected. It is interesting, however, that the most well-characterized miRNA involved in hematopoiesis and blood cancers were not represented [119] in the significant DE set. This supports the idea that the massive control of miRNA expression by BORIS could be related to limiting the impact of BORIS activation.

The most dramatic effect among highly expressed known miRNA was observed for let-7a-3p, as well as: let-7i-5p, miR-1296-3p, miR-340-5p, miR-3184-3p, miR-7-5p, miR-142-5p, and miR-224-3p (Supplementary Table S4). The let-7i-5p has been placed in the molecular makeup of CML as one of several MiRs targeting NFKB1 [89]. The miR-1296-3p is known to play an tumor suppressing and inhibitory role in cancer [120, 121], as well as repress the expression of chromosome maintenance genes that are upregulated in tumors [122]. The higher levels of miR-340-5p are known to induce apoptosis [123]. miR-3184-3p is on the opposite strand relatively to the intron in the enclosing protein coding gene, and its antisense, miR-423-3p, is notably downregulated (Supplementary Table S4), indicating that BORIS mediated control is exerted differentially dependent on the direction of the gene. Incidentally, the miR-423-3p upregulation is known to promote proliferation of tumor cells [124, 125], while it’s inhibition has an opposite effect [126]. miR-7-5p is encoded at three different loci in human genome, none of which appear to be associated with BORIS binding in cis. Nevertheless, it is known to be p53-dependent [127], and its expression was shown to inhibit cancer cells [128, 129]. miR-142-5p has been shown to induce apoptosis in non-blood cancer cells [130] and inhibit cell cycle *via* beta-catenin/WNT signaling [131]. It has been also shown to be a component of macrophage-specific signaling program [132].

Thus, in general, it is evident that BORIS controls multiple species of miRNA, with a substantial spectrum of functions. This control is exerted through a number of mechanisms, from the whole locus-control functions to individual promoter occupancy of the host genes. This phenomenon may act as a buffer for the ability of BORIS to act as a repressor and activator of multiple genes, thus limiting the scope of BORIS activity, despite abundant expression.

In addition to miRNA, it was also revealed that piRNAs, which, when expressed in cancers, essentially qualify as non-protein-coding CT genes, are also regulated by BORIS. The degree, to which this control is direct, remains to be investigated further, as the knowledge of individual piRNAs’ regulation is extremely limited. Incidentally, while some piRNAs (such as piR-17033), and including most of the novel species, have originated from the endogenous retrovirus (ERV) sequences, none were mapped within or in the vicinity of SVA elements, reinforcing our original conclusion that BORIS directly regulates SVA by binding at SVA VNTRs [36].

Furthermore, while we could hypothesize that BORIS is positively regulating piRNA expression in testis, that mode of regulation appears to be distorted in cancer cells, as multiple piRNA are upregulated upon BORIS KD. The involvement of BORIS in piRNA expression, at least in cancer cells, is a significant finding pertaining to BORIS biology in cancers. It could be directly related to the genomic instability in cancers expressing BORIS, due to the uncoupling of piRNA expression control from the normal somatic mechanisms.

In conclusion, it is evident that, while BORIS strongly binds promoter areas and TSS of thousands of genes in K562 cells, the resulting set of DE expressed genes upon the substantial depletion of BORIS is rather limited. It could be a reflection of the corresponding “buffering” role of multiple DE miRNAs that are concurrently regulated by BORIS. Nevertheless, this is a strong indication that the key to BORIS functions in cancer, and particularly to K562 addiction to BORIS, could be narrowed down to a tangible set of factors, rather than attributed to the whole-epigenome remodeling in a germline-like fashion.

## MATERIALS AND METHODS

### Cell culture and generation of stable inducible knockdown cell lines

K562 cells and stably transfected derivatives were grown in IMDM (Hyclone) with 10% or 20% Tet-On certified FBS. BORIS knock-down vectors were constructed to express either of the two shRNA templates, GGAAATACCACGATGCAAATT (Site 1) or GGTGTGAAATGCTCCTCAACA (Site 2). To construct the vectors, the synthetic double-strand oligonucleotides were inserted into AgeI and EcoRI restriction sites of the pLKO-Tet-On-neo vector. To package lentivirus in HEK293T/17 cells they were co-transfected with the Tet-pLKO-Neo [133] (Addgene) vector (vector-only control) or with the same vector expressing either of two alternative anti-BORIS shRNA together with two packaging plasmids psPAX2 and Pmd2.G. The stocks of lentivirus were harvested 72 hr after the transfection. K562 cells grown to 40%-50% confluence were infected with 500µl of lentivirus stock using 8µg/ml polybrene (Sigma). Culture media supplemented with 600µg/ml G418 was replaced 12 hr after infection, and cells were selected for 4 weeks or more. Over ten independent stable cell lines expressing anti-BORIS shRNA were generated. For each, cells resistant to G418 were plated into in 96-well plates, and individual wells were analyzed by RT-qPCR and anti-BORIS immunoblotting. The wells that showed robust BORIS downregulation were pooled from one plate and frozen. For BORIS knockdown, stably infected cells were induced with 200ng/ml doxycycline resulting in the activation of the Tet-On promoter driving the expression of shRNAs.

### Quantitative RT-PCR

Total RNA from cultured cells was extracted using Trizol (Invitrogen). cDNA was synthesized with the PrimescriptTM RT Reagent Kit with genomic DNA Eraser (Perfect Real Time) from TaKaRa. Quantitative PCR (qPCR) was done with the SYBR Premix Ex Taq (TaKaRa) on the Mx30005P qPCR workstation (Agilent).

### Immunoblotting

For whole-cell protein extracts, cells were washed with PBS with 1X protease inhibitor cocktail (Roche Applied Science) and then lysed with SDS-PAGE sample buffer. Protein extracts were separated by SDS-PAGE and transferred to a PVDF membrane, followed by the incubation with the primary and then secondary antibodies fused to fluorochromes for LI-COR Odyssey (LI-COR Biosciences) detection and quantification.

The anti-BORIS antibodies were as described in [33]. Anti alpha-tubulin Abs were from Merck (Sigma Aldrich). Anti-CA2, anti-AKR1B1, and anti-SOX6 Abs were from Abcam.

### RNA-seq analysis

For the RNA-seq, the inducible BORIS KD and empty vector control K562 transgenic cell lines were used. To induce BORIS KD, doxycycline was applied for 96 hr resulting in a reduction of BORIS mRNA level 2.5-4.5 fold, with the corresponding decrease of BORIS protein levels. At least 4 biological replicates were analyzed for each set of experimental conditions. For all NGS mRNA analyses, the inducible stable shRNA K562 cells were plated at ∼40% confluence in DMEM media and induced in the presence of doxycycline (200 ng/ml) for 96 hr; the cells were then harvested and frozen. mRNA was extracted, libraries were prepared and sequenced at RiboBio (Guangzhou) using Illumina HiSeq3000. The initial assessment of quality of RNA was at the Agilent Genomics 2200 TapeStation. For the library quality testing, Agilent 2100 Bioanalyzer and ABI StepOnePlus Real-TimePCR System were used.

On average, 20 million reads were obtained from each experiment. The results of RNA-seq experiments were analyzed using a conventional pipeline. They were first analyzed for consistency and reproducibility, aligned to the human reference genome (hg38) using TopHat2 with the parameters —segment-length 20 -G GRCh38, then processed using Cufflinks 2.0.0 [134] (parameter settings: -g GRCh38), then Cuffmerge (-p 8 -o -g GRCh38 -s) and Cuffdiff (-p 8 -L -b -M, loci over 500k filtered out). The set of significantly changed DE genes set was then additionally filtered to remove low expression genes with expression < 0.5 FPKM. Expressed loci with no assigned gene symbol were also excluded from further analysis.

For ncRNA NGS analyses, the RNA preparations were done by BGI (Shenzhen) and sequenced using Illumina HiSeq4000. Different size RNA segments were separated by PAGE gel with 18-30nt stripe selected. Then, after the adapter ligation and RT-PCR procedures were recovered by PAGE gel and recycled products dissolved in the EB solution.

The resulting 49nt tags were cleaned from low quality tags and adaptor contaminants, afterwards they were categorized with all the small tags failed to fall into known category to be grouped as potentially novel miRNA with the following priority assumption: miRNA > piRNA > snoRNA > Rfam > other sRNA (Thus, each RNA falls into a single unique category). Additionally, for potential novel miRNAs their ability to form characteristic hairpin structure together with the Dicer cleavage site and the minimum free energy were analyzed *via* miRDeep2 [135]. Target prediction analysis with GO enrichment and KEGG [136] pathway analysis were made.

For piRNA prediction, the Piano [137] tool was used. To discover potential piRNAs [138], their structural features (namely, the fact, that each strand of this 22-24nt long dual-strand RNA is 2nt longer on the 3’ end than the paired one) were tested by aligning tags to each other.

To map the reads, either Bowtie2 [139] or cmsearch [140] (mapping to Rfam) were used with the following parameters: -q -L 16 —phred64 -p 6 (Bowtie2) and —cpu 6 —noali (cmsearch). The expression levels were calculated as TPM (transcripts per kb, million) [141]. For possible target prediction, RNAhybrid [142], miRanda [143] and TargetScan [144] software tools were used with the following parameters: -en -20 -strict (miRanda); -b 100 -c -f 2,8 -m 100000 -v 3 -u 3 -e -20 -p 1 -s 3utr_human (RNAhybrid). DESs screening and mapping were performed by using DEGseq [145] and pheatmap software, respectively.

The mRNA datasets were deposited at NCBI GEO with the accession number GSE99825, and the sRNA datasets and the corresponding metadata were deposited at NCBI GEO with the accession number GSE99899. Both datasets are accessible together through the series’ accession number GSE99900.

## SUPPLEMENTARY MATERIALS FIGURE AND TABLES













## Abbreviations

Differential expression / differentially expressed: (DE)
cancer-testis: (CT)
zinc fingers: (ZF)
chronic myeloid leukemia: (CML)
knockdown: (KD)
knockout: (KO)
empty lentivirus vector: (EV)
breakpoint cluster region: (BCR)
the cancer genome atlas: (TCGA)
chromatin immuno-precipitation: (ChIP)
chromatin interaction analysis by paired-end tag/sequencing: (ChIA-PET)
transcription start site(s): (TSS)
micro RNA: (miRNA, MiR)
Piwi-interacting RNA: (piRNAs)
transposable element(s): (TE)
long noncoding RNAs: (lncRNAs)
endogenous retrovirus: (ERV)
SINE VNTR ALU element: (SVA)
variable number tandem repeat(s): (VNTR)
transcript reads per kilobase per million: (TPM)
fragments per kilobase of transcript per million fragments mapped: (FPKM)
