# Supplementary material for: The downregulation of putative anticancer target BORIS/CTCFL in an addicted myeloid cancer cell line modulates the expression of multiple protein coding and ncRNA genes
Source: Oncotarget. 2017 Sep 2;8(43):73448–68. doi: 10.18632/oncotarget.20627 (PMC5650274; doi:10.18632/oncotarget.20627)
Supplement: Supplementary file 1 [file oncotarget-08-73448-s001.pdf]

The downregulation of putative anticancer target BORIS/CTCFL in an addicted myeloid cancer cell line modulates the expression of multiple protein coding and ncRNA genes

SUPPLEMENTARY MATERIALS

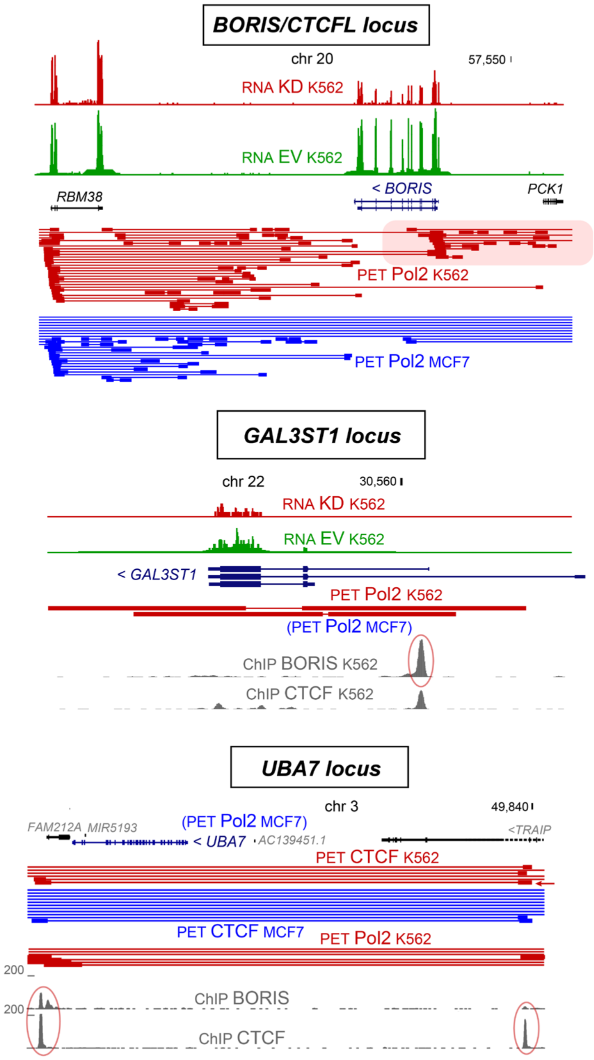

Supplementary Figure 1: Examples of DE genes in a typical BORIS KD experiment. The *BORIS* locus panel shows the decrease of RNA signal (TopHat alignment) for BORIS upon KD versus EV, while two flanking genes remain unaffected. The ChIA pet maps from [46] show that there is distinct set of BORIS-specific interactions (shaded) in K562 (red), while BORIS-negative cell line MCF7 (blue) has none of those. The similarly arranged *GAL3ST1* locus panel shows the relative decrease of *GAL3ST1* RNA signal upon KD. The ChIA pet maps from [46] show that there are locus-specific interactions in BORIS-positive K562 (red), while BORIS-negative cell line MCF7 has none. The lower panel displays the ChIP data from [33] showing BORIS binding at the *GAL3ST1*promoter. Coordinates are shown in kb, all the comparable panes are in the same scale. The genomic structure of *UBA7* locus is aligned with the ChIA-PET data is from [46] and the ChIP signal, which shows the two putative associated sites for CTCF and BORIS (circled).

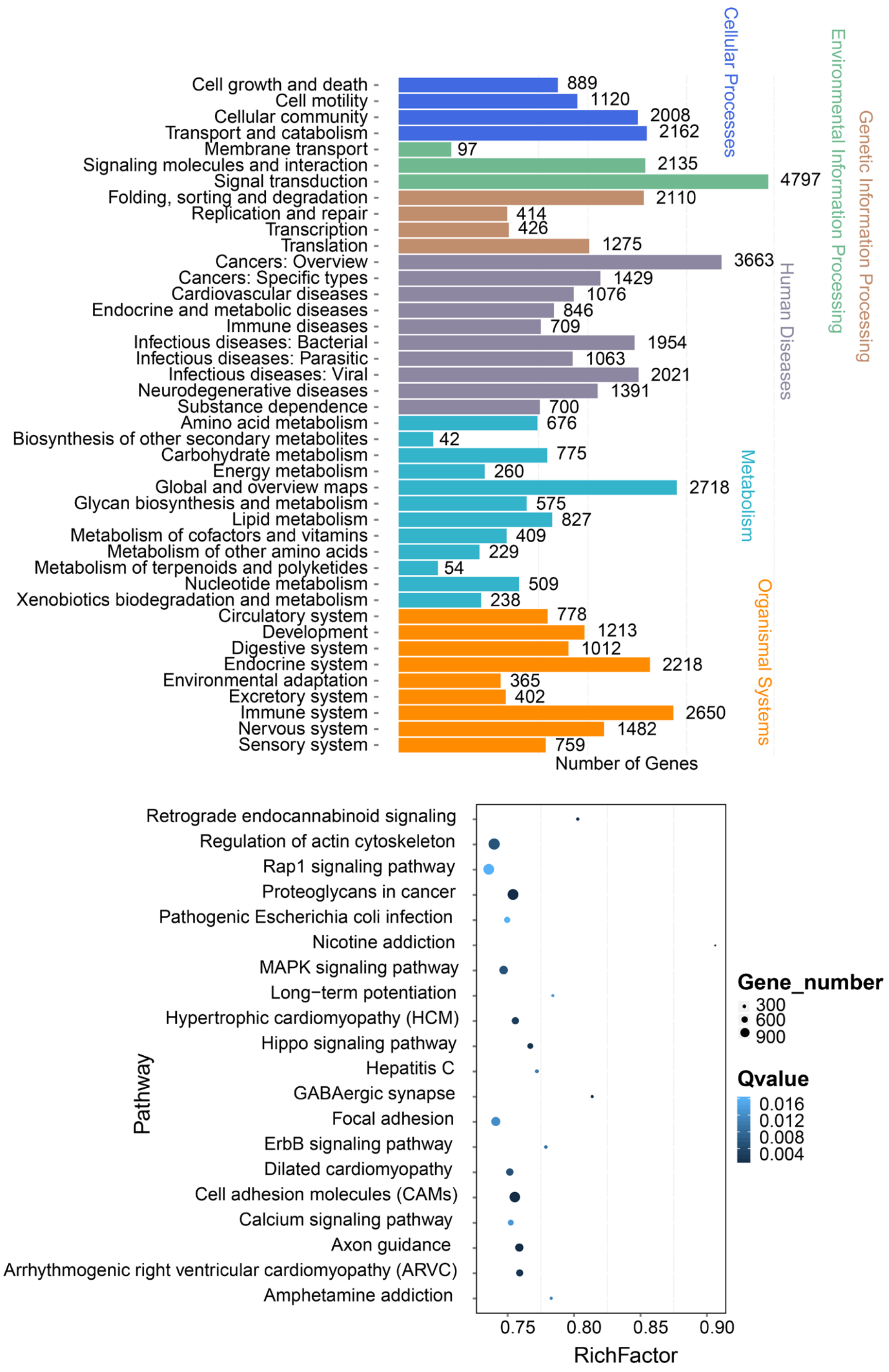

Supplementary Figure 2: KEGG and pathway enrich analyses among the targets of DE miRNAs.

For Supplementary Tables see in Supplementary Files
